# Magnetism and the Trimeron Bond

**DOI:** 10.1021/acs.chemmater.2c00275

**Published:** 2022-03-30

**Authors:** J. Paul Attfield

**Affiliations:** Centre for Science at Extreme Conditions and School of Chemistry, University of Edinburgh, Mayfield Road, Edinburgh, EH9 3JZ United Kingdom

## Abstract

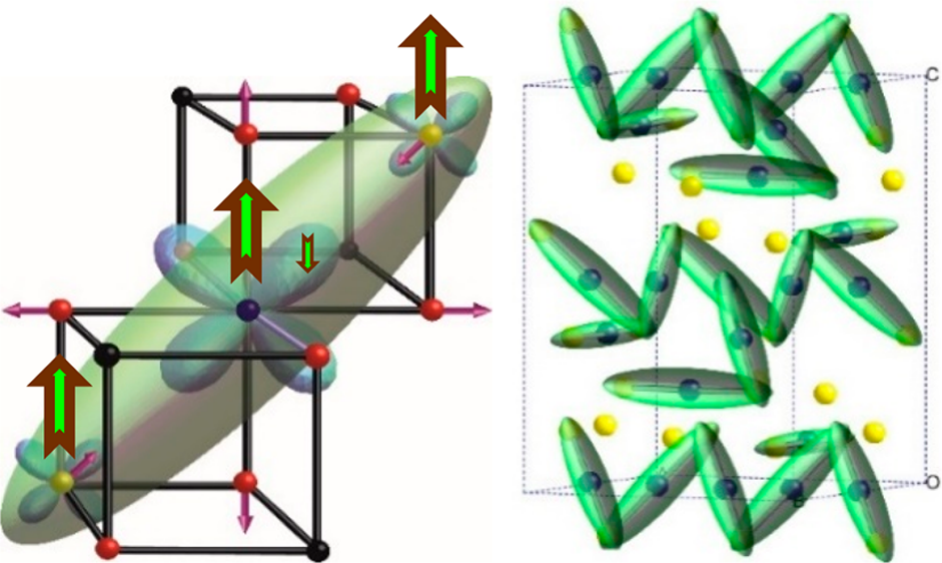

A review of progress in understanding the Verwey transition in magnetite (Fe_3_O_4_) over the past decade is presented. This electronic and structural transition at *T*_V_ ≈ 125 K was reported in 1939 and has since been a contentious issue in magnetism. Long range Fe^2+^/Fe^3+^ charge ordering has been confirmed below the transition from crystal structure refinement, and Fe^2+^ orbital ordering and formation of trimerons through weak bonding of Fe^2+^ states to two Fe neighbors has been discovered. This model has accounted for many spectroscopic observations such as the ^57^Fe NMR frequencies. The trimeron lifetime has been measured, and trimeron soft modes have been observed. The origin of the first to second order crossover of Verwey transitions in doped magnetites has been revealed by a nanoparticle study. Electronic and structural fluctuations are found to persist to temperatures far above *T*_V_ and local structural distortions track the bulk magnetization, disappearing at the 850 K Curie transition. New binary mixed-valent iron oxides discovered at high pressure are found to have electronic transitions and orbital molecule ground states similar to those of magnetite.

## Introduction

Goodenough’s seminal “Magnetism and the Chemical Bond” introduced important concepts such as orbital-based superexchange rules for explaining magnetism in solids.^1^ The magnetic behavior of many transition metal compounds was rationalized using these rules, including the ferrimagnetism of the original magnetic material magnetite (Fe_3_O_4_), and related ferrite spinels. However, magnetite also undergoes a change in properties at *T*_V_ ≈ 125 K reported by Verwey in 1939 that reveals further electronic complexity at low temperatures.^2^ The full low temperature crystal structure was determined in 2012 and revealed direct magnetically driven Fe–Fe bonding interactions within three-center trimeron units.^3^ This paper will review progress on the long-running effort to understand the Verwey transition of magnetite made over the subsequent decade.

At ambient temperature, magnetite adopts the cubic spinel-type structure with an inverse formal charge distribution Fe^3+^[Fe^2.5+^]_2_O_4_ over tetrahedral A and octahedral B sites, shown throughout as A[B]_2_O_4_. Ferrimagnetic order occurs below the Curie transition at *T*_C_ ≈ 850 K as there are twice as many up-spins at the B sites as there are down-spins at the A sites. Each cation site has a Fe^3+^ 3*d*^5^*S* = 5/2 core spin, and rapid hopping of the one extra down-spin electron for every two B sites results in minority-spin-polarized electronic conductivity so all B-sites are structurally and spectroscopically equivalent. The Verwey transition at *T*_V_ ≈ 125 K, where magnetite undergoes a structural distortion and becomes electrically insulating, is observed in measurements of heat capacity, conductivity, magnetization, and many other properties. Progress made on understanding this transition during the 20th Century is covered in an extensive review by Walz.^4^

Verwey proposed that the 125 K transition is driven by an ordering of Fe^2+^ and Fe^3+^ ions at the B-sites equivalent to localization of the minority spin extra electrons,^2^ a phenomenon now known as charge ordering that has been verified in many other oxides.^5^ However, initial simple charge ordered models were incompatible with crystallographic data, and a complex lattice distortion to a monoclinic √2 × √2 × 2 superstructure of the cubic room temperature spinel lattice was later identified.^6,7^ The supercell has *Cc* space group symmetry and contains 56 symmetry-unique atoms (compered to three in the cubic *Fd*3̅*m* high temperature cell). The complexity of this acentric superstructure in addition to practical difficulties arising from microtwinning of *Cc* domains below the Verwey transition hampered single crystal diffraction studies of the low temperature structure. Several partial structure refinements using powder diffraction data with symmetry constraints,^8−10^ or Fe K-edge resonant X-ray diffraction studies,^11−13^ reported some evidence for charge order during 2001–2011.

**Figure 1:**
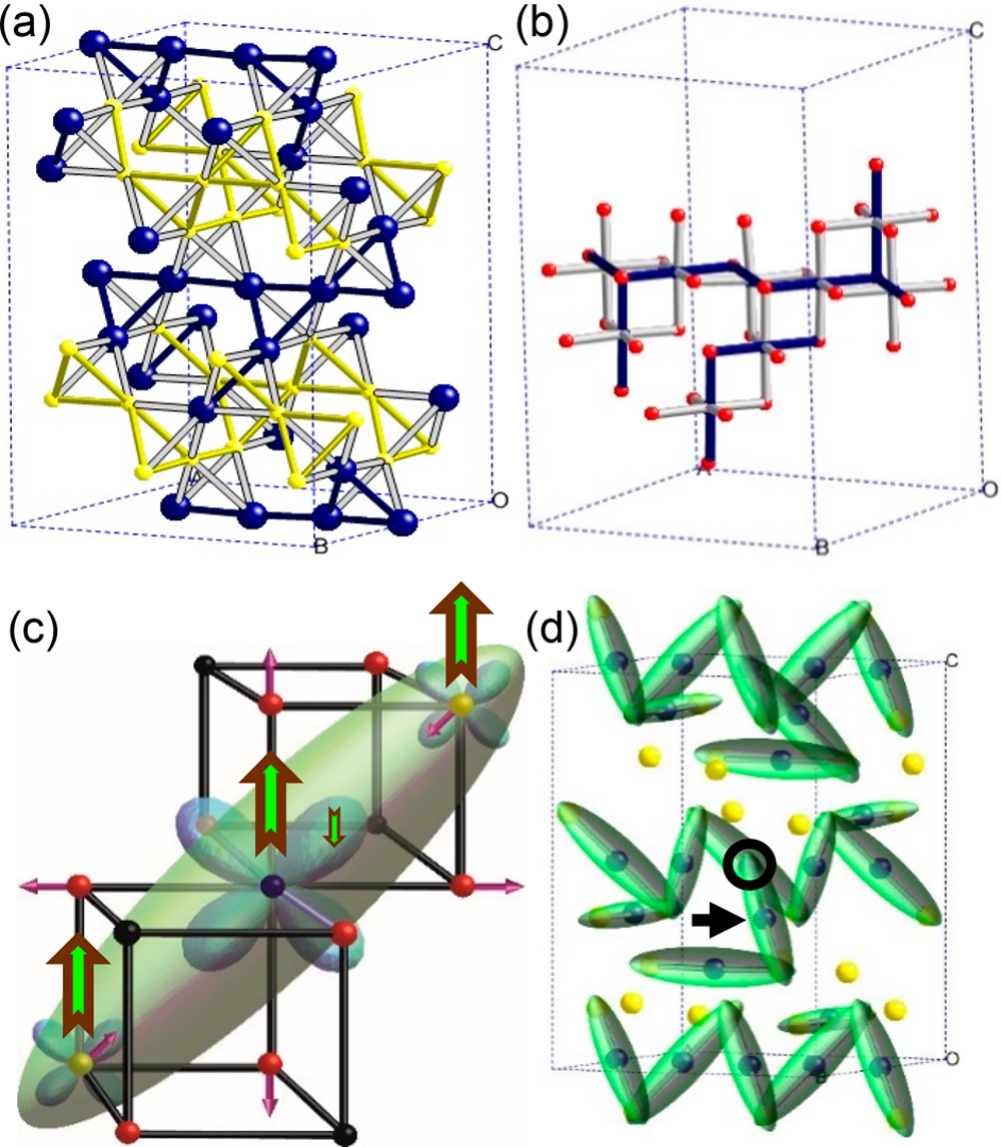
Charge, orbital, and trimeron orders in the low temperature *Cc* supercell of magnetite, as deduced from the SWA refinement model.^3^ (a) Distribution of Fe^2+^/Fe^3+^ charge states (blue/yellow spheres). (b) Compressive tetragonal Jahn–Teller distortions arising from orbital order within a single Fe^2+^ chain shown as long/short bonds (gray/blue lines) to oxygen atoms (red spheres). (c) Single trimeron unit consisting of three Fe sites with parallel *S* = 5/2 spins as shown by the up brown-green arrows. Orbital order at the central Fe^2+^ site localizes an antiparallel spin electron (small down arrow) in one of the *t*_2*g*_ orbitals which distorts the local structure through elongation of four Fe–O bonds and shortening of the Fe–Fe distances through weak bonding to two Fe neighbors in the same plane, as indicated by the purple arrows. The down spin electron density is approximated by the green ellipsoid. (d) Trimeron distribution in the low temperature magnetite structure, with Fe^2+^/Fe^3+^ shown as blue/yellow spheres. Most trimerons have charge configuration Fe^3+^–Fe^2+^–Fe^3+^, but one has Fe^2+^–Fe^2+^–Fe^3+^; the terminating Fe^2+^ is circled. This trimeron is selectively destroyed in Fe_2.98_Zn_0.02_O_4_ as the arrowed Fe^2+^ site is preferentially oxidized.^53^ Material reprinted with permission from ref (53). Copyright 2012, Nature Publishing Group, a division of Macmillan Publishers Limited.

A full refinement of the low temperature *Cc* superstructure of magnetite against microcrystal synchrotron diffraction data recorded at 90 K was reported by Senn, Wright, and Attfield (hereafter the SWA model).^3^ Analysis of the local distortion modes of the BO_6_ octahedra revealed complex patterns of Fe^2+^/Fe^3+^ charge ordering and Fe^2+^*t*_2*g*_-orbital ordering evidenced by compressive tetragonal Jahn–Teller distortions, as shown respectively in Figure 1a,b. A later ellipsoidal analysis of local coordinations in the SWA model also revealed the charge and orbital ordering features.^14^ However, additional structural displacements leading to anomalous shortening of some B–B distances showed that the extra down-spin electrons are not fully localized as Fe^2+^ states but are instead spread over linear three-site units where weak magnetically driven Fe–Fe–Fe bonding results in highly structured three-site polarons termed “trimerons” (Figure 1c). The low temperature structure can thus also be described as a network of corner sharing trimerons (Figure 1d). It is notable that, out of many theoretical predictions made prior to publication of the SWA model, one computational study did correctly predict the charge and orbital ordering patterns within the *Cc* superstructure of magnetite and also reported some of the trimeron distortions.^15^

This paper will review progress on magnetite and the Verwey transition over the past decade, showing how experimental and computational results have been used to test and build upon the charge, orbital, and trimeron orderings and other features of the SWA model and describing new iron oxides that have been discovered to have trimeron-based and related ground states. The review is organized into sections that cover insights into (A) the low and (B) the high temperature states of magnetite (below and above *T*_V_); (C) results for off-stoichiometric and cation-doped magnetites; and (D) discoveries of other iron oxides with trimeron-based and related ground states.

## Results

### A Low Temperature Magnetite (below *T*_V_)

The experimental reproducibility of the SWA model for the *Cc* superstructure was verified by a subsequent study in which 22 high-accuracy structure refinements using synchrotron X-ray data from three different 10–40 μm grains of magnetite were performed at temperatures from 20 to 124 K.^16^ Analysis of the coordinates showed little variation across the models except for small thermal changes at temperatures just below *T*_V_.

The low temperature *Cc* crystal structure is complex and difficult to visualize, and so it is useful to represent the 168 independent shifts in (*x*, *y*, *z*) atomic coordinates as 168 equivalent frozen phonon amplitudes. Only one O atom mode is present in the high temperature cubic structure as a static distortion, and the rest all freeze at the Verwey transition. A total of 80 modes are required for the closest centric description (preserving inversion symmetry) in space group *C*2/*c*, and an additional 88 are needed for the full acentric *Cc* description. The 168 modes belong to four classes; Γ, Δ, X, and W point distortions. The magnitudes of all 168 modes in the SWA model have been analyzed,^3,17^ and their thermal variations were also reported.^16^ Differences between the amplitudes of centric and acentric branches of Δ, X, and W modes were all found to contribute to the significant off-center atomic distortions in the *Cc* magnetite structure that can lead to ferroelectric and multiferroic properties. It would be convenient to be able to describe the *Cc* magnetite structure in terms of a few frozen phonon modes, but no good approximation is yet apparent, although brief details of an attempt at mode parametrization are reported.^18^

Further diffraction evidence for charge order in the *Cc* phase of magnetite has come from a resonant multiwave X-ray diffraction study.^19^ The use of three-wave diffraction intensities corrected for self-absorption effects that may have affected earlier studies, giving clear evidence for charge ordering at the B-sites in agreement with the SWA model.

Electronic DFT band structure calculations of the *Cc* magnetite structure have been reported using the SWA model positions^20,21^ or with relaxed coordinates.^22^ These have confirmed the reported charge and orbital orderings and show that the extra electrons occupy a narrow minority-spin band just below the Fermi level. Real space plots of the electron density show a buildup of charge between Fe atoms that form trimeron units, consistent with a weak bonding effect.^20^ Interplay between the orbital order and spin–orbit coupling was found to account for the reported magnetoelectric effect in the *Cc* structure.^21^

**Figure 2:**
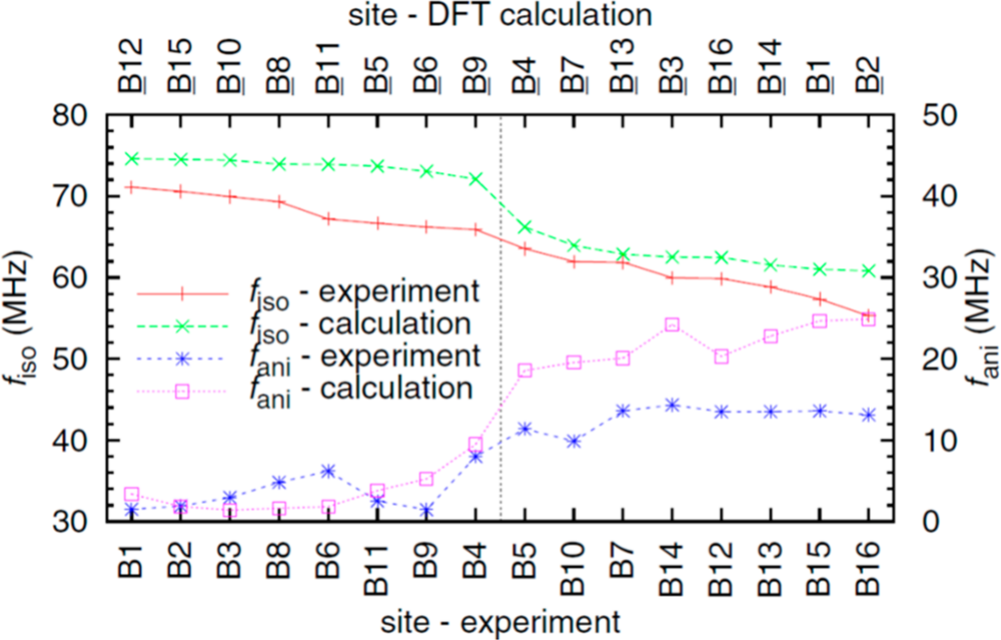
Comparison of the anisotropic and isotropic parts of the ^57^Fe NMR frequencies for the 16 unique octahedral B sites in the low temperature structure of magnetite. Experimental data and site labels are from ref (23). The eight sites to the left/right of the broken line correspond to Fe^3+^/Fe^2+^ states. Calculated DFT results are from ref (25) with sites numbered in the order that they appear in ref (3). Reprinted with permission from ref (25). Copyright 2015 by the American Physical Society.

DFT calculations have also been used to investigate how well the SWA model accounts for spectroscopic observations of the low temperature magnetite structure. ^57^Fe NMR is particularly important as it is the only noncrystallographic technique to have resolved signals from all 24 unique Fe atoms (at 8 A sites and 16 B sites) within the *Cc* cell.^23^ Hyperfine fields from DFT calculations were used to compute the ^57^Fe resonance frequencies,^24,25^ and these are in excellent agreement with reported values as shown for the B sites in Figure 2. These calculations also support the trimeron description as ref (25) notes “the hyperfine anisotropy data obtained from the DFT calculations support the trimeron concept as the central Fe^2+^-like ions of the suggested trimerons exhibit significantly larger anisotropy than the end ions ... in agreement with expectations deduced from the description of the electron distribution in the trimerons”.

The ^57^Fe Mössbauer spectrum has also been simulated using hyperfine parameters from DFT calculations based on the SWA model.^26^ Approximation to four sextets was found to give good agreement with Mössbauer data from a high quality single crystal of magnetite. The four signals are in an 8:8:5:3 ratio to account respectively for 8 A-site Fe^3+^ ions, 8 B-site Fe^3+^ ions, 5 B-site Fe^2+^ ions where the extra electron occupies *d*_*xz*_ or *d*_*yz*_ orbitals, and 3 B-site Fe^2+^ ions with the extra electron in the *d*_*xy*_ orbital. The latter group is distinct as the *d*_*xy*_ orbitals lie perpendicular to the magnetization in the *Cc* structure giving rise to lower effective magnetic fields and larger electric field gradients, and they also have distinctive NMR frequencies.^24,25^ Further Mössbauer and resonant X-ray experiments have suggested that trimeron direction changes around a fixed central Fe^2+^ ion when the easy magnetization axis of the *Cc* phase is switching by an applied magnetic field.^27^ Recent phonon calculations for the *Cc* structure have shown good agreement with inelastic neutron, X-ray, and nuclear scattering data, revealing strong trimeron–phonon coupling, especially for trimerons oriented parallel to the axes of the monoclinic *Cc* cell.^28^

Dynamics of the low temperature phase of magnetite have been explored using coherent and other light sources. The lifetime of individual trimerons was measured in a pump–probe experiment where the effects of femtosecond laser excitation were followed by soft X-ray diffraction.^29^ This found that metallization of the low temperature state of magnetite proceeds in two steps. Initial trimeron destruction takes place in 300 fs, with phase segregation into metallic and insulating regions following on an ∼1500 fs time scale. A full study of the photoinduced phase segregation through optical conductivity measurements was subsequently reported.^30^ Soft electronic modes of the trimeron order were recently revealed by low temperature optical pump–terahertz probe experiments.^31^ These modes show critical softening and so are associated with the Verwey transition, and they most likely correspond to the sliding of trimerons along their long axes.

The Verwey transition is suppressed at a pressure of 8 GPa as confirmed by changes in elastic constants observed in a high pressure study.^32^ Pronounced elastic anisotropies in acoustic waves along the cubic-[110] direction were attributed to the presence of the long Fe–Fe–Fe trimeron axis parallel to this direction. The large shape strain at the Verwey transition makes the low temperature phase sensitive to nonhydrostatic stresses, and twin populations are altered.^33^ Uniaxial stresses are found to increase *T*_V_ initially as twin orientations with higher *T*_V_’s become favored. Twinning of the *Cc* structure is eliminated in small particles, and a study of magnetite nanocrystal size effects showed that the Verwey transition is decreased slightly to *T*_V_ ≈ 120 K at a 20 nm particle size and is fully suppressed in particles below 6 nm.^34^ This demonstrates that the minimum coherence distance for the bulk long-range electronic order is around the length of 10 trimerons.

### B High Temperature Magnetite (above *T*_V_)

Above the Verwey transition at *T*_V_ ≈ 125 K, magnetite has the cubic spinel-type structure in space group *Fd*3̅*m* with formal charge distribution Fe^3+^[Fe^2.5+^]_2_O_4_ at ambient temperature. A high temperature powder neutron diffraction study revealed changes in the thermal expansion coefficient and variable oxygen coordinate near 700 K that were attributed to the onset of charge transfer between the tetrahedral A and octahedral B sites.^35^ This has been confirmed by recent X-ray spectroscopy measurements which showed that charge transfer from B to A sites, represented by *x* in the formula Fe^3+^_1–*x*_Fe^2+^_*x*_[Fe^3+^_1+*x*_Fe^2+^_1–*x*_]O_4_, starts near 330 K and increases up to *x* = 0.125 at 840 K near *T*_C_.^36^ Migration of Fe cations from octahedral sites to tetrahedral vacancies was reported at higher temperatures.

A key question has been whether disordered charge, orbital, and trimeron correlations persist in the high-temperature cubic phase. Observation of diffuse scattering just above *T*_V_ shows that local structural correlations are present, and a single crystal X-ray experiment revealed highly structured diffuse scatter that persists to at least 300 K.^37^ This has been corroborated by inelastic scattering studies of the lattice vibrations. Raman studies have shown that changes in vibrational modes associated with the Verwey transition occur from *T*_V_ up to ∼200 K,^38,39^ and an inelastic neutron scattering study up to 293 K found discontinuities in transverse acoustic phonons at *T*_V_ and a decoupling of electronic and phonon dynamics consistent with slow fluctuations of trimerons in the cubic phase.^40^ Anomalous broadening of Δ and X mode phonons up to at least 293 K was reported from an inelastic X-ray scattering (IXS) study.^41^

Resonant IXS (RIXS) has been used to explore electronic excitations of the octahedral Fe cations in cubic magnetite, revealing magnetic excitations driven by polaronic distortions that persist to at least 550 K.^42^ Other RIXS experiments have shown that that the orbital components of the magnetic moments are ordered noncollinearly at 300 K, consistent with dynamic distortions associated with polaron formation.^43^ However, a RIXS-MLD (magnetic linear dichroism) experiment at 170 K revealed that the polarization dependence of the spin–orbital excitations is incompatible with the purely tetragonal Jahn–Teller distortions of the ideal trimeron quasiparticle (Figure 1c) and suggested that trigonal distortions may be more relevant.^44^

**Figure 3:**
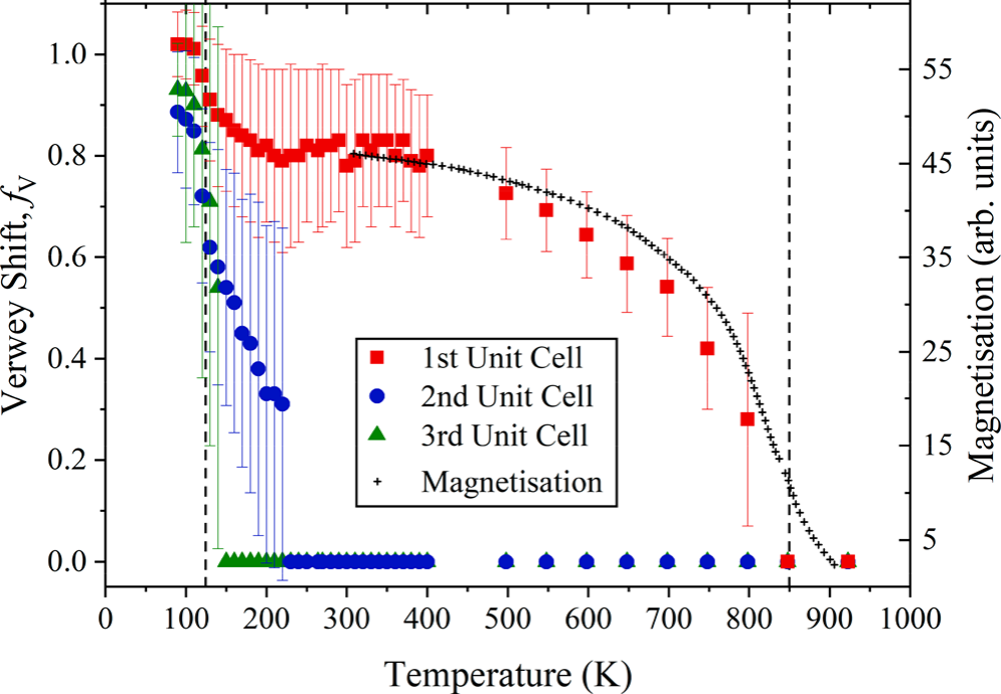
Thermal variations of local structural displacements due to electronic fluctuations in magnetite measured from below the Verwey transition at *T*_V_ ≈ 125 K to above the Curie point at *T*_C_ ≈ 850 K.^46^ Displacements are quantified by the Verwey shift parameter *f*_V_ which is normalized to the average atomic shift at 90 K in the SWA model. *f*_V_ was fitted to first/second/third unit cell ranges of the X-ray PDF, which describe short/medium/long-range electronic orders. The first unit cell values show that substantial local structural distortions persist up to *T*_C_ and closely match the reported variation of the bulk magnetization.^47^ This demonstrates that the structural and electronic fluctuations responsible for the Verwey transition are a direct result of the long-range magnetic order. Material reprinted with permission from ref (46). Published 2019 by Springer Nature Limited under a Creative Commons license (http://creativecommons.org/licenses/by/4.0/).

Analysis of the PDF (pair distribution function) derived from total scattering experiments has been used to evidence local structural distortions within the cubic phase of magnetite. Room temperature X-ray and neutron PDFs of a nanoparticle magnetite sample were found to be not fitted well by the cubic *Fd*3̅*m* structure, and lower symmetry space groups were used to model the local distortions.^45^ The SWA model was used to fit the average degree of local distortion over short, medium, and long-range length scales in an X-ray PDF study covering a wide range of temperatures (90–923 K).^46^ The resulting plots in Figure 3 show that long-range structural distortions fall sharply to zero just above *T*_V_, while medium range distortions persist up to 250–300 K, which matches Raman observations of modes associated with the electronic order.^38,39^ However, short-range structural correlations, on the length scale of an individual trimeron, remain present above *T*_V_ and decrease to zero near the Curie transition at *T*_C_ ≈ 850 K, following a similar temperature dependence to the reported bulk magnetization.^47^ This also matches the thermal transfer of extra electrons (Fe^2+^ states) from octahedral to tetrahedral sites seen by X-ray spectroscopy.^36^ The weak bonding Fe–Fe interactions in a trimeron require ferromagnetic alignment of the three core *S* = 5/2 spins so that the extra minority spin electron can be delocalized over the three Fe ions, as shown in Figure 1c. Hence, magnetization is coupled to local Fe displacements to which the X-ray PDF is particularly sensitive. Fe cation displacements due to Fe–Fe bonding interactions emerging below *T*_C_ were thus identified as the primary driver of the local structural distortions that give rise to the Verwey transition in magnetite.

### C Doped Magnetites

Many cations can be substituted into magnetite to generate the cubic spinel family of ferrites. Comparison of room temperature X-ray and neutron PDFs for MFe_2_O_4_ (M = Mn, Fe, Co, and Ni) spinel nanoparticles showed that the M = Mn, Co, and Ni dopants suppressed the local distortions observed for M = Fe magnetite,^45^ consistent with loss of the Fe^2+^ states associated with local charge, orbital, and trimeron orders.

**Figure 4:**
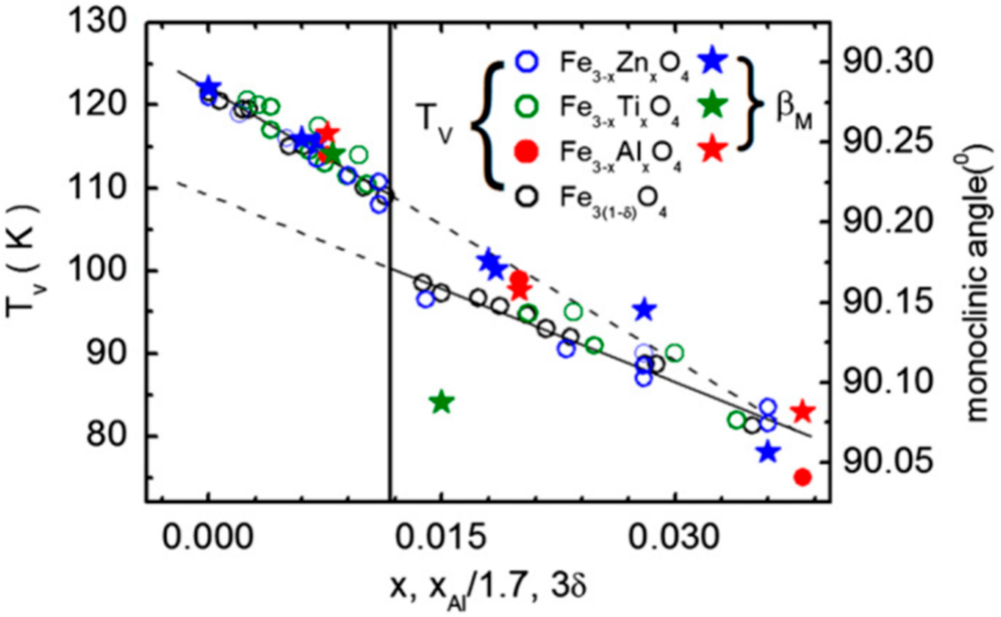
Variations of *T*_V_ and the monoclinic angle of the low temperature *Cc* cell with doping parameters for nonstoichiometric and cation-doped magnetites in ref (49). The break between first and second order regimes of the Verwey transition is marked by the vertical line. Reprinted with permission from ref (49). Copyright 2012 Elsevier.

Magnetite has a small intrinsic range of nonstoichiometry due to iron-deficiency as Fe_3(1−δ)_O_4_ up to 3δ ≈ 0.035. Studies of nonstoichiometric and lightly cation-doped magnetites showed that *T*_V_ is suppressed by doping, and a change from sharp first order to broad second order Verwey transitions was reported around hole doping of *x* = 3δ = 0.012^48,49^ as shown in Figure 4. The lattice distortion associated with formation of the low temperature *Cc* state is observed in both first and second order regimes, and no change in phonon spectra between the regimes was found in nuclear inelastic scattering experiments.^50^ Zn^2+^ substitutes at the tetrahedral A sites and so provides a clean way to hole-dope the B-cation sites as Fe^3+^_1–*x*_Zn^2+^_*x*_[Fe^3+^_1+*x*_Fe^2+^_1–*x*_]O_4_, and detailed characterization of Fe_3–*x*_Zn_*x*_O_4_ samples by Mössbauer spectroscopy and X-ray diffraction has been reported.^51^

**Figure 5:**
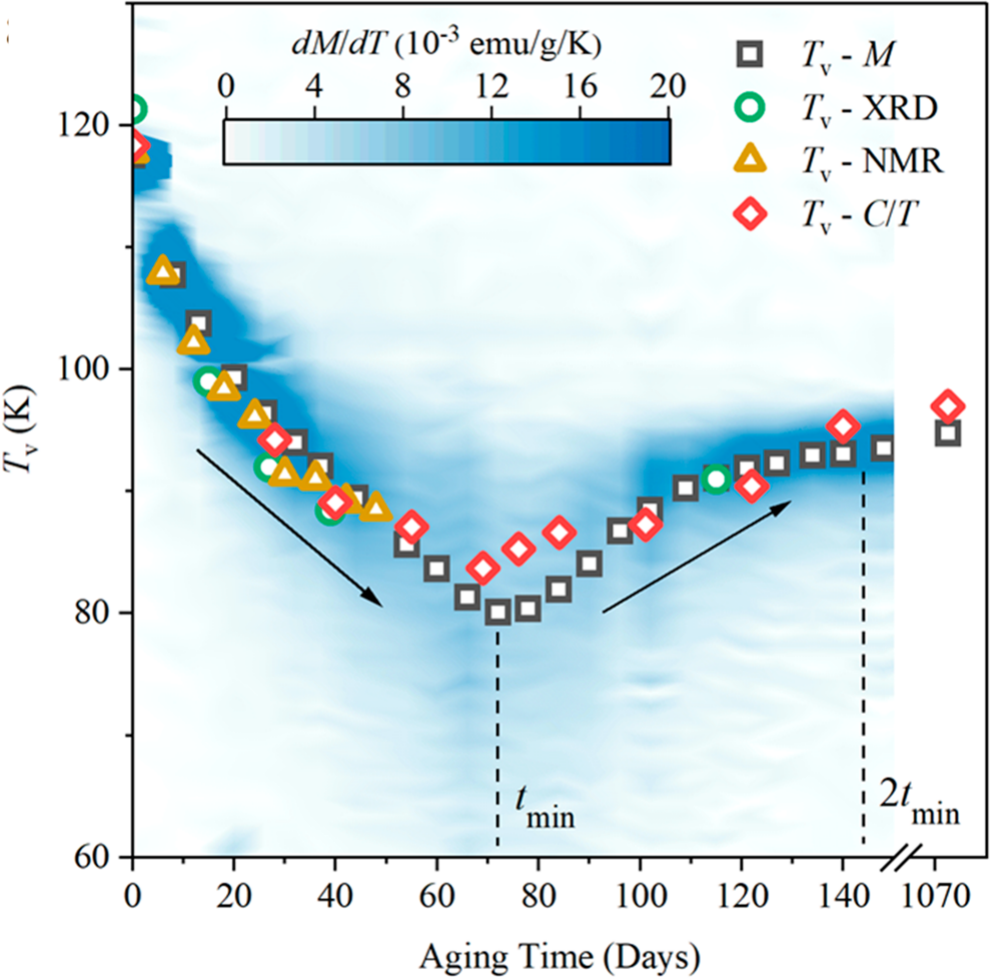
Variation of the Verwey transition of magnetite nanoparticles with oxidation time in air from ref (52). Square, circle, triangle, and diamond symbols show *T*_V_ values from magnetization (*M*), powder X-ray diffraction (XRD), NMR, and heat capacity (*C*/*T*) measurements, respectively. The dashed line indicates the time *t*_min_, at which the minimum of *T*_V_ is observed in the magnetization data. The color density represents the temperature derivative of the magnetization, d*M*/d*T*, which shows how the Verwey transition broadens around *t*_min_ and later sharpens as the persistent *T*_V_ = 95 K value is reached near 2*t*_min_. This persistent value is identified as the limit of the first order (homogeneous) doping regime. Material reprinted from ref (52). Published 2021 by Springer Nature Limited under a Creative Commons license (http://creativecommons.org/licenses/by/4.0/).

Insight into the origin of the first and second order regimes of doped magnetites has recently been provided by a study of slow oxidation of magnetite nanoparticles.^52^ This revealed that the Verwey transition is initially suppressed to a minimum value at *T*_V_ ≈ 80 K, but on further oxidation recovers to a persistent value of *T*_V_ = 95 K as shown in Figure 5. This variation demonstrates that the Verwey transition is suppressed not only by the doping effect from the added oxygen but also by inhomogeneous strains from the concentration gradient developed between the oxygen-rich exterior and oxygen-poor interior of the nanoparticles during oxidation, and this was confirmed by quantitative modeling. Observation of the persistent value of *T*_V_ = 95 K close to the *T*_V_ ≈ 100 K crossover between first and second-order Verwey transitions (Figure 4)^47,48^ shows that the crossover corresponds to the intrinsic lower temperature limit of the Verwey transition in homogeneously doped magnetite. Lower *T*_V_ values down to 70 K in the second-order regime result from additional effects of strain gradients on the transition. Hence, the reported critical doping δ_c_ = 0.0039 at the crossover^48^ is identified as the true upper limit for homogeneous oxygen doping of magnetite.

Low temperature structure refinements of several doped magnetites have been carried out using the same microcrystal method as in ref (3). All of these found the same monoclinic *Cc* lattice distortion as in pure magnetite. Refinements for a Fe_3(1−δ)_O_4_ material with estimated hole-doping of 3δ = 0.0116,^53^ and for a natural mineral sample of composition Fe_2.986_Al_0.007_Si_0.003_Mg_0.002_Mn_0.002_O_4_^54^ both gave coordinates similar to those of the SWA model, with the same charge, orbital, and trimeron orders apparent, although with some blurring of the local electronic distortions. However, the refinement of a more heavily doped Fe_3–*x*_Zn_*x*_O_4_ structure with estimated *x* = 0.0228 found a remarkable suppression of charge, orbital, and trimeron features at one of the eight Fe^2+^ sites within the *Cc* cell.^53^ This site is unique in having its trimeron terminated by another Fe^2+^ cation, as shown in Figure 1d, and thus was reported as having a lower ionization potential due to electron–electron repulsion. This discovered doping selectivity is remarkable as it corresponds to a “charge order within a charge order” where the rest of the charge, orbital, and trimeron network of magnetite remains robust while one site is preferentially oxidized.

### D Other Iron Oxides

The trimerons observed in the low temperature structure of magnetite are an example of orbital molecules, clusters made up of coupled orbital states on several metal ions within an orbitally ordered (and often also charge ordered) solid. Further examples of orbital molecules are found in other transition metal compounds, e.g., the V_2_ dimers formed below the metal–insulator transition in VO_2_, and are reviewed elsewhere.^55^ Recent discoveries of trimeron and related dimeron cluster orders in iron oxides are described below.

**Figure 6:**
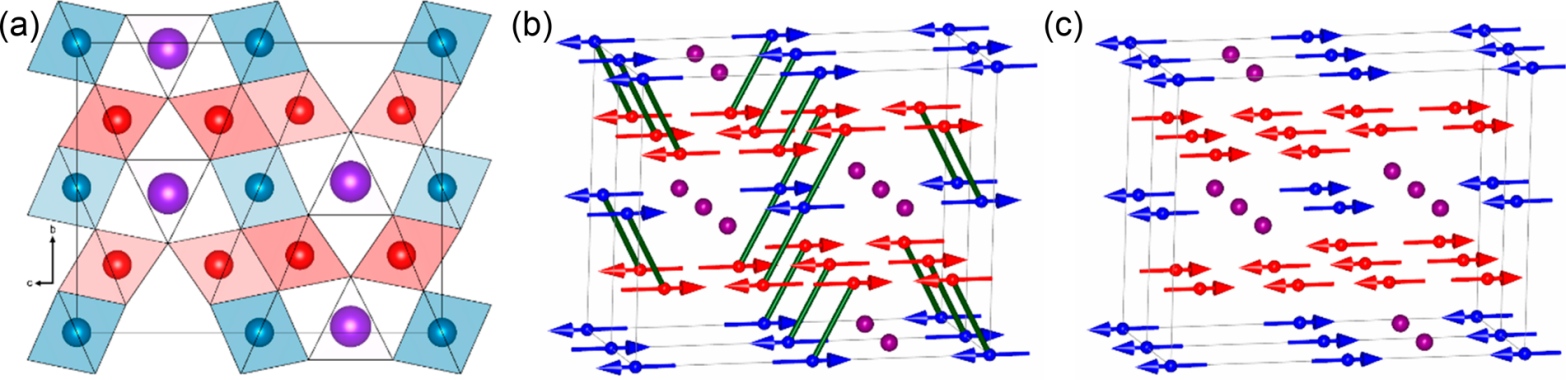
(a) Projection of the Fe_4_O_5_ structure type.^56^ This has two inequivalent types of FeO_6_ octahedra (red and blue) and Fe^2+^ ions within trigonal prismatic tunnels (purple). The latter are replaced by Ca^2+^ in CaFe_3_O_5_. Electronic phase separation in lightly doped CaFe_3_O_5_ leads to a mixture of (b) a charge ordered (CO) ground state, where groups of three spins have ferromagnetic alignment leading to Fe^3+^–Fe^2+^–Fe^3+^ charge order and trimeron formation (green lines), and (c) a charge averaged (CA) phase where the spins in the same groups are antiferromagnetically coupled and no charge or trimeron order is observed.^68,69^ Material from ref (68), http://creativecommons.org/licenses/by/4.0/.

Fe_3_O_4_ was previously the only known stoichiometric, binary, mixed-valent iron oxide, but the past decade has seen an explosion of iron oxide discoveries. The breakthrough occurred when geophysicists exploring possible new iron oxides formed at high pressures and temperatures within Earth’s mantle discovered a new composition, Fe_4_O_5_ (Figure 6a), that could be recovered to ambient conditions.^56^ This has led to discoveries of new binary mixed-valent iron oxides falling into the Fe_*n*_O_*n*+1_ (*n* = 4 and 5) and Fe_*m*_O_*m*+2_ (*m* = 5 and 7) homologous series.^57^ The Fe_*n*_O_*n*+1_ materials show Verwey-type transitions with charge, orbital, and orbital molecule ordering in their ground states.

Fe_4_O_5_ orders antiferromagnetically below ∼320 K and shows a Verwey-type charge ordering transition at 150 K and a further spin canting transition at 85 K.^58^ The Fe^2+^/Fe^3+^ charge ordered structure is incommensurate and consists of trimerons and also dimerons. “Dimeron” is used to describe two-center units analogous to trimerons, where one extra electron is shared between two neighboring B site cations with parallel *S* = 5/2 spins, giving a symmetric Fe_2_^5+^ dimer. Two further high pressure charge ordered phases, one containing dimerons and trimerons and the second based on dimerons alone, have subsequently been discovered from exploration of the low temperature phase diagram up to 50 GPa.^59^ Fe_5_O_6_ undergoes a Verwey-type charge ordering transition at 275 K leading to dimeron ordering, with long-range antiferromagnetic order of the dimerons below 100 K, and the dimeron order is reported to be stable to at least 20 GPa.^60^

Electronic ordering has also been reported in several ternaries derived from magnetite or the above new iron oxides. High pressure Mössbauer, conductivity, and diffraction studies of the warwickite type oxoborate BFe_2_O_4_ (Fe_2_OBO_3_), which is charge ordered without orbital molecule formation below 280 K at ambient pressure,^61^ reported formation of an electron-localized dimeron phase from 16 GPa up to at least 50 GPa.^62,63^ CaFe_5_O_7_, the Ca-stabilized version of the as-yet unreported *n* = 6 member of the Fe_*n*_O_*n*+1_ family, is reported to show a magnetic transition at 360 K accompanied by a monoclinic lattice distortion,^64^ although possible charge and orbital molecule orders are not yet established.

Charge ordering has been reported in MFe_3_O_5_ derivatives of Fe_4_O_5_ for M = Mn and Ca. Spins in MnFe_3_O_5_ order antiferromagnetically below 350 K with further spin transitions at 150 and 60 K. The latter is driven by charge ordering of Fe^2+^ and Fe^3+^ but without apparent orbital molecule formation.^65,66^ CaFe_3_O_5_ orders magnetically near 300 K, forming commensurate and incommensurate charge, orbital, and trimeron ordered (CO) phases when stoichiometric,^67^ and is notable for displaying electronic phase separation into CO and charge averaged (CA) ground states when slightly doped.^68,69^ Direct comparison of local distortions and spin orders from neutron-refined crystal and magnetic structures of coexisting CO and CA phases in a slightly off-stoichiometric Ca_0.96_Fe_3.04_O_5_ sample provides a clear demonstration of the conditions for trimeron order (Figure 6b,c).^68^ The CO phase has ferromagnetic spin order and Fe^3+^–Fe^2+^–Fe^3+^ charge ordering across groups of three edge-sharing FeO_6_ octahedra, with Jahn–Teller compression at the central Fe^2+^ site and shortening of the Fe–Fe distances. All of these observations are consistent with trimeron formation. However, the CA phase has antiferromagnetic up–down–up spin order across the same sites, with absence of charge and orbital ordering distortions and Fe–Fe shortening, all demonstrating that trimerons are not present.

## Discussion

The published results described above show that very significant progress in understanding the Verwey transition of magnetite has been made over the past decade. Much of this has been enabled by technique developments. In particular, state-of-the art X-ray synchrotron beamlines have been used for microcrystal determinations of the *Cc* ground state crystal structure,^3,16,53,54^ resonant and multiwave diffraction studies,^19,27^ nuclear inelastic scattering,^50^ ambient and high pressure powder diffraction studies,^32,45^ diffuse and total scattering (PDF) experiments,^37,45,46^ X-ray absorption spectroscopy and MCD,^36^ and resonant and nonresonant IXS,^41−44^ with the majority of these experiments performed at the ESRF. Coherent light sources have also been important for pump–probe studies of lattice dynamics.^29,31^ Improved DFT codes have supported many of these investigations, also enabling accurate NMR and Mössbauer spectra to be simulated.^24−26,28^ Developments in nanoparticle chemistry have enabled influences of particle size and oxygen content on the Verwey transiton to be determined in exquisite detail.^34,52^ High pressure discoveries of new mixed-valent binary iron oxides have broadened insights into magnetite.^56,58−60^

The SWA model for the *Cc* crystal structure of magnetite below the Verwey transition has been corroborated by subsequent microcrystal diffraction studies showing only small changes with temperature or doping, although the selective destruction of one trimeron in Fe_2.98_Zn_0.02_O_4_ is a notable structural variation.^53^ The Fe^2+^/Fe^3+^ charge ordering and Fe^2+^*t*_2*g*_-orbital ordering deduced from the SWA model have been confirmed by many other techniques, notably through spectroscopic assignment of all 24 ^57^Fe NMR signals and the four classes of Mössbauer resonances.^24−26^ The long-running hypothesis that magnetite has a charge ordered ground state, originally proposed by Verwey in 1939, has thus been comprehensively confirmed over the past decade. The origin of the change from first to second order Verwey transitions in doped magnetites has also been revealed by a nanoparticle oxidation study.^52^

The trimeron interpretation of the low temperature electronic order has also been supported by subsequent studies; a critical test was again the ^57^Fe NMR spectrum where the trimeron model was shown to give a better description than an alternative orbital picture.^25^ Measurement of the trimeron lifetime as a distinct step in the photon-induced metallization of magnetite^29^ and recent observation of trimeron soft modes^31^ add further weight. Comparison of trimeron and nontrimeron ground states in phase separated CaFe_3_O_5_ gives direct observation of the conditions for trimeron formation.^68,69^ Electronic DFT band structure calculations have confirmed the charge, orbital, and trimeron orderings in magnetite^20−22^ and have enabled quantitative interpretation of many spectroscopic results. A recent study (ref (28)) concluded that their “results indicate the validity of trimerons (and trimeron–phonon coupling) to explain the physics of magnetite much beyond their original formulation”, suggesting that further insights may derive from more sophisticated future theoretical treatments of trimeron quasiparticles.

Understanding of the low temperature state of magnetite has assisted interpretation of the high temperature cubic phase. RIXS has been particularly insightful in showing that charge and orbital fluctuations remain active far above *T*_V_.^42^ Two studies have revealed similar variations in local distortions^46^ and B to A site charge transfer^36^ as temperature increases toward *T*_C_ ≈ 850 K. These paint a consistent picture that long-range magnetic order creates the trimeron bonding distortions that drive charge and orbital ordering, thereby suppressing B to A site charge transfer. Critical fluctuations in the magnetization as temperature increases toward *T*_C_ thus lead to loss of the local structural distortions and the onset of intersite charge transfer.

However, studies of local structure in the cubic phase of magnetite have not definitively shown that trimeron distortions are responsible for the Fe and other displacements, and one RIXS study suggests that this geometry is not dominant.^44^ It is notable that several other mixed valent Fe oxides (Fe_4_O_5_,^58,59^ Fe_5_O_6_,^60^ and Fe_2_OBO_3_)^62,63^ show ordering of two-site dimerons or a mix of trimeron and dimeron units, at ambient or high pressure. Hence, the cubic phase of magnetite might contain mixtures of one-site (single Fe^2+^ ions), two-site (dimeron), three-site (trimeron), and perhaps other orbital molecule fluctuations that are likely to change their populations with temperature. The nature of magnetite at ambient temperature (above *T*_V_) thus remains a continuing topic for human inquiry, as it has for around 3000 years.

## Conclusions

Discoveries over the past decade have led to great progress in understanding of the Verwey transition at *T*_V_ ≈ 125 K in magnetite. Long range Fe^2+^/Fe^3+^ charge ordering below the transition is confirmed from full refinement of the acentric *Cc* crystal structure, and Fe^2+^ orbital ordering and formation of trimerons through weak bonding of Fe^2+^ states to two Fe neighbors have been discovered. This model has accounted for many spectroscopic observations such as the ^57^Fe NMR frequencies. The trimeron lifetime has been measured, and trimeron soft modes have been observed. The origin of the first to second order crossover of Verwey transitions in doped magnetites has also been revealed by a nanoparticle oxidation study. Studies of the cubic phase of magnetite have shown that electronic and structural fluctuations persist to temperatures far above *T*_V_ and local structural distortions track the bulk magnetization, disappearing at the Curie transition at *T*_C_ ≈ 850 K. However, whether the high-temperature structural fluctuations are trimeron-like remains to be determined. New binary mixed-valent iron oxides discovered at high pressure are found to have similar electronic transitions and orbital molecule ground states, establishing a broader context for the electronic properties of magnetite.

## References

[ref1] Goodenough J. B. Magnetism and the Chemical Bond; Interscience Publishers: New York, London, 1963.

[ref2] Verwey E. J. W. Electronic conduction of magnetite (Fe_3_O_4_) and its transition at low temperatures. Nature 1939, 144, 327–328. 10.1038/144327b0.

[ref3] Senn M. S.; Wright J. P.; Attfield J. P. Charge order and three-site distortions in the Verwey structure of magnetite. Nature 2012, 481, 173–176. 10.1038/nature10704. 22190035

[ref4] Walz F. The Verwey transition – a topical review. J. Phys.: Condens. Matter 2002, 14, R285–R340. 10.1088/0953-8984/14/12/203.

[ref5] Attfield J. P. Charge ordering in transition metal oxides. Solid State Sci. 2006, 8, 861–867. 10.1016/j.solidstatesciences.2005.02.011.

[ref6] Yoshida J.; Iida S. X-ray diffraction study on the low temperature phase of magnetite. J. Phys. Soc. Jpn. 1977, 42, 230–237. 10.1143/JPSJ.42.230.

[ref7] Iizumi M.; Koetzle T. F.; Shirane G.; Chikazumi S.; Matsui M.; Todo S. Structure of magnetite (Fe3O4) below the Verwey transition temperature. Acta Cryst. B 1982, 38, 2121–2133. 10.1107/S0567740882008176.

[ref8] Wright J. P.; Attfield J. P.; Radaelli P. G. Long range charge ordering in magnetite below the Verwey transition. Phys. Rev. Lett. 2001, 87, 266401 10.1103/PhysRevLett.87.266401. 11800847

[ref9] Wright J. P.; Attfield J. P.; Radaelli P. G. Charge ordered structure of magnetite below the Verwey transition. Phys. Rev. B 2002, 66, 214422 10.1103/PhysRevB.66.214422.

[ref10] Blasco J.; Garcia J.; Subias G. Structural transformation in magnetite below the Verwey transition. Phys. Rev. B 2011, 83, 104105 10.1103/PhysRevB.83.104105.

[ref11] Goff R. J.; Wright J. P.; Attfield J. P.; Radaelli P. G. Resonant x-ray diffraction study of the charge ordering in magnetite. J. Phys.: Condens. Matter 2005, 17, 7633 10.1088/0953-8984/17/48/015.

[ref12] Nazarenko E.; Lorenzo J. E.; Joly Y.; Hodeau J. L.; Mannix D.; Marin C. Resonant X-Ray Diffraction Studies on the Charge Ordering in Magnetite. Phys. Rev. Lett. 2006, 97, 056403 10.1103/PhysRevLett.97.056403. 17026123

[ref13] Joly Y.; Lorenzo J. E.; Nazarenko E.; Hodeau J.-L.; Mannix D.; Marin C. Low-temperature structure of magnetite studied using resonant x-ray scattering. Phys. Rev. B 2008, 78, 134110 10.1103/PhysRevB.78.134110.

[ref14] Cumby J.; Attfield J. P. Ellipsoidal analysis of coordination polyhedra. Nat. Commun. 2017, 8, 14235 10.1038/ncomms14235. 28146146 PMC5296646

[ref15] Yamauchi K.; Fukushima T.; Picozzi S. Ferroelectricity in multiferroic magnetite Fe3O4 driven by noncentrosymmetric Fe2+/Fe3+ charge-ordering: First-principles study. Phys. Rev. B 2009, 79, 212404 10.1103/PhysRevB.79.212404.

[ref16] Senn M. S.; Wright J. P.; Cumby J.; Attfield J. P. Charge localization in the Verwey structure of magnetite. Phys. Rev. B 2015, 92, 024104 10.1103/PhysRevB.92.024104.

[ref17] Senn M. S.; Wright J. P.; Attfield J. P. The verwey phase of magnetite — a long-running mystery in magnetism. J. Kor. Phys. Soc. 2013, 62, 1372–1375. 10.3938/jkps.62.1372.

[ref18] Perez-Mato J.; Kocsis B.; Tasci E.; Aroyo M. Mode parameterization of structures with very low symmetry: PZT and magnetite. Acta Cryst. A 2014, 70, C501–C501. 10.1107/S2053273314094984.

[ref19] Weng S. C.; Lee Y. R.; Chen C. G.; Chu C. H.; Soo Y. L.; Chang S. L. Direct Observation of Charge Ordering in Magnetite Using Resonant Multiwave X-Ray Diffraction. Phys. Rev. Lett. 2012, 108, 146404 10.1103/PhysRevLett.108.146404. 22540813

[ref20] Senn M. S.; Loa I.; Wright J. P.; Attfield J. P. Electronic orders in the Verwey structure of magnetite. Phys. Rev. B 2012, 85, 125119 10.1103/PhysRevB.85.125119.

[ref21] Yamauchi K.; Picozzi S. Orbital degrees of freedom as origin of magnetoelectric coupling in magnetite. Phys. Rev. B 2012, 85, 085131 10.1103/PhysRevB.85.085131.

[ref22] Liu H. S.; Di Valentin C. Band Gap in Magnetite above Verwey Temperature Induced by Symmetry Breaking. J. Phys. Chem. C 2017, 121, 25736–25742. 10.1021/acs.jpcc.7b09387. PMC5706067 29201266

[ref23] Mizoguchi M. Charge and Orbital Ordering Structure of Fe3O4 in the Low-Temperature Phase as Deduced from NMR Study. J. Phys. Soc. Jpn. 2001, 70, 2333–2344. 10.1143/JPSJ.70.2333.

[ref24] Patterson C. H. Hybrid DFT calculation of Fe-57 NMR resonances and orbital order in magnetite. Phys. Rev. B 2014, 90, 075134 10.1103/PhysRevB.90.075134.

[ref25] Reznicek R.; Chlan V.; Stepankova H.; Novak P. Hyperfine field and electronic structure of magnetite below the Verwey transition. Phys. Rev. B 2015, 91, 125134 10.1103/PhysRevB.91.125134.

[ref26] Reznicek R.; Chlan V.; Stepankova H.; Novak P.; Zukrowski J.; Kozlowski A.; Kakol Z.; Tarnawski Z.; Honig J. M. Understanding the Mossbauer spectrum of magnetite below the Verwey transition: Ab initio calculations, simulation, and experiment. Phys. Rev. B 2017, 96, 195124 10.1103/PhysRevB.96.195124.

[ref27] Kolodziej T.; Bialo I.; Tabis W.; Zubko M.; Zukrowski J.; Latka K.; Lorenzo J. E.; Mazzoli C.; Kakol Z.; Kozlowski A.; Tarnawski Z.; Wilke E.; Babik P.; Chlan V.; Reznicek R.; Stepankova H.; Novak P.; Joly Y.; Niewolski J.; Honig J. M. Magnetic field induced structural changes in magnetite observed by resonant x-ray diffraction and Mossbauer spectroscopy. Phys. Rev. B 2020, 102, 075126 10.1103/PhysRevB.102.075126.

[ref28] Piekarz P.; Legut D.; Baldini E.; Belvin C. A.; Kolodziej T.; Tabis W.; Kozlowski A.; Kakol Z.; Tarnawski Z.; Lorenzana J.; Gedik N.; Oles A. M.; Honig J. M.; Parlinski K. Trimeron-phonon coupling in magnetite. Phys. Rev. B 2021, 103, 104303 10.1103/PhysRevB.103.104303.

[ref29] de Jong S.; Kukreja R.; Trabant C.; Pontius N.; Chang C. F.; Kachel T.; Beye M.; Sorgenfrei F.; Back C. H.; Braeuer B.; Schlotter W. F.; Turner J. J.; Krupin O.; Doehler M.; Zhu D.; Hossain M. A.; Scherz A. O.; Fausti D.; Novelli F.; Esposito M.; Lee W. S.; Chuang Y. D.; Lu D. H.; Moore R. G.; Yi M.; Trigo M.; Kirchmann P.; Pathey L.; Golden M. S.; Buchholz M.; Metcalf P.; Parmigiani F.; Wurth W.; Foehlisch A.; Schuessler-Langeheine C.; Duerr H. A. Speed limit of the insulator-metal transition in magnetite. Nat. Mater. 2013, 12, 882–886. 10.1038/nmat3718. 23892787

[ref30] Randi F.; Vergara I.; Novelli F.; Esposito M.; Dell’Angela M.; Brabers V. A. M.; Metcalf P.; Kukreja R.; Duerr H. A.; Fausti D.; Grueninger M.; Parmigiani F. Phase separation in the nonequilibrium Verwey transition in magnetite. Phys. Rev. B 2016, 93, 054305 10.1103/PhysRevB.93.054305.

[ref31] Baldini E.; Belvin C. A.; Rodriguez-Vega M.; Ozel I. O.; Legut D.; Kozlowski A.; Oles A. M.; Parlinski K.; Piekarz P.; Lorenzana J.; Fiete G. A.; Gedik N. Discovery of the soft electronic modes of the trimeron order in magnetite. Nat. Phys. 2020, 16, 541–545. 10.1038/s41567-020-0823-y.

[ref32] Lin J. F.; Wu J. J.; Zhu J.; Mao Z.; Said A. H.; Leu B. M.; Cheng J. G.; Uwatoko Y.; Jin C. Q.; Zhou J. S. Abnormal Elastic and Vibrational Behaviors of Magnetite at High Pressures. Sci. Rep. 2015, 4, 6282 10.1038/srep06282. PMC4153994 25186916

[ref33] Coe R. S.; Egli R.; Gilder S. A.; Wright J. P. The thermodynamic effect of nonhydrostatic stress on the Verwey transition. Earth Planet. Sci. Lett. 2012, 319–320, 207–217. 10.1016/j.epsl.2011.11.021.

[ref34] Lee J.; Kwon S. G.; Park J.-G.; Hyeon T. Size Dependence of Metal–Insulator Transition in Stoichiometric Fe3O4 Nanocrystals. Nano Lett. 2015, 15, 4337–4342. 10.1021/acs.nanolett.5b00331. 26079048

[ref35] Levy D.; Giustetto R.; Hoser A. Structure of magnetite (Fe3O4) above the Curie temperature: a cation ordering study. Phys. Chem. Min. 2012, 39, 169–176. 10.1007/s00269-011-0472-x.

[ref36] Elnaggar H.; Graas S.; Lafuerza S.; Detlefs B.; Tabiś W.; Gala M. A.; Ismail A.; van der Eerden A.; Sikora M.; Honig J. M.; Glatzel P.; de Groot F. Temperature-Driven Self-Doping in Magnetite. Phys. Rev. Lett. 2021, 127, 186402 10.1103/PhysRevLett.127.186402. 34767399

[ref37] Bosak A.; Chernyshov D.; Hoesch M.; Piekarz P.; Le Tacon M.; Krisch M.; Kozlowski A.; Oles A. M.; Parlinski K. Short-Range Correlations in Magnetite above the Verwey Temperature. Phys. Rev. X 2014, 4, 011040 10.1103/PhysRevX.4.011040.

[ref38] Baghaie Yazdi M.; Choi K.-Y.; Wulferding D.; Lemmens P.; Alff L. Raman study of the Verwey transition in magnetite thin films. New J. Phys. 2013, 15, 103032 10.1088/1367-2630/15/10/103032.

[ref39] Borroni S.; Teyssier J.; Piekarz P.; Kuzmenko A. B.; Oles A. M.; Lorenzana J.; Carbone F. Light scattering from the critical modes of the Verwey transition in magnetite. Phys. Rev. B 2018, 98, 184301 10.1103/PhysRevB.98.184301.

[ref40] Borroni S.; Tucker G. S.; Pennacchio F.; Rajeswari J.; Stuhr U.; Pisoni A.; Lorenzana J.; Ronnow H. M.; Carbone F. Mapping the lattice dynamical anomaly of the order parameters across the Verwey transition in magnetite. New J. Phys. 2017, 19, 103013 10.1088/1367-2630/aa83a3.

[ref41] Hoesch M.; Piekarz P.; Bosak A.; Le Tacon M.; Krisch M.; Kozlowski A.; Oles A. M.; Parlinski K. Anharmonicity due to Electron-Phonon Coupling in Magnetite. Phys. Rev. Lett. 2013, 110, 207204 10.1103/PhysRevLett.110.207204. 25167445

[ref42] Huang H. Y.; Chen Z. Y.; Wang R.-P.; de Groot F. M. F.; Wu W. B.; Okamoto J.; Chainani A.; Singh A.; Li Z.-Y.; Zhou J.-S.; Jeng H.-T.; Guo G. Y.; Park J. G.; Tjeng L. H.; Chen C. T.; Huang D. J. Jahn-Teller distortion driven magnetic polarons in magnetite. Nat. Commun. 2017, 8, 15929 10.1038/ncomms15929. 28660878 PMC5493765

[ref43] Elnaggar H.; Sainctavit Ph.; Juhin A.; Lafuerza S.; Wilhelm F.; Rogalev A.; Arrio M.-A.; Brouder Ch.; van der Linden M.; Kakol Z.; Sikora M.; Haverkort M. W.; Glatzel P.; de Groot F. M. F. Noncollinear Ordering of the Orbital Magnetic Moments in Magnetite. Phys. Rev. Lett. 2019, 123, 207201 10.1103/PhysRevLett.123.207201. 31809079

[ref44] Elnaggar H.; Wang R.; Lafuerza S.; Paris E.; Komarek A. C.; Guo H.; Tseng Y.; McNally D.; Frati F.; Haverkort M. W.; Sikora M.; Schmitt T.; de Groot F. M. F. Possible absence of trimeron correlations above the Verwey temperature in Fe3O4. Phys. Rev. B 2020, 101, 085107 10.1103/PhysRevB.101.085107.

[ref45] Deepak F. L.; Banobre-Lopez M.; Carbo-Argibay E.; Cerqueira M. F.; Pineiro-Redondo Y.; Rivas J.; Thompson C. M.; Kamali S.; Rodriguez-Abreu C.; Kovnir K.; Kolen’ko Y. V. A Systematic Study of the Structural and Magnetic Properties of Mn-, Co-, and Ni-Doped Colloidal Magnetite Nanoparticles. J. Phys. Chem. C 2015, 119, 11947–11957. 10.1021/acs.jpcc.5b01575.

[ref46] Perversi G.; Pachoud E.; Cumby J.; Hudspeth J. M.; Wright J. P.; Kimber S. A. J.; Attfield J. P. Co-emergence of magnetic order and structural fluctuations in magnetite. Nat. Commun. 2019, 10, 2857 10.1038/s41467-019-10949-9. 31253806 PMC6599026

[ref47] Ponomar V. P.; Dudchenko N. O.; Brik A. B. Thermal stability of micro- and nanoscale magnetite by thermomagnetic analysis data. 2017 IEEE 7th International Conference Nanomaterials: Application & Properties (NAP), Odessa, 2017; 02MFPM03-1, 10.1109/NAP.2017.8190407.

[ref48] Honig J. M. Analysis of the Verwey transition in magnetite. J. Alloys Compd. 1995, 229, 24–39. 10.1016/0925-8388(95)01677-5.

[ref49] Kąkol Z.; Owoc D.; Przewoźnik J.; Sikora M.; Kapusta C.; Zając D.; Kozłowski A.; Sabol J. E.; Honig J. M. The effect of doping on global lattice properties of magnetite Fe3-xMexO4 (Me = Zn, Ti and Al). J. Solid State Chem. 2012, 192, 120–126. 10.1016/j.jssc.2012.04.001.

[ref50] Kołodziej T.; Kozłowski A.; Piekarz P.; Tabiś W.; Kąkol Z.; Zając M.; Tarnawski Z.; Honig J. M.; Oleś A. M.; Parlinski K. Nuclear inelastic scattering studies of lattice dynamics in magnetite with a first- and second-order Verwey transition. Phys. Rev. B 2012, 85, 104301 10.1103/PhysRevB.85.104301.

[ref51] Chlan V.; Zukrowski J.; Bosak A.; Kakol Z.; Kozłowski A.; Tarnawski Z.; Reznicek R.; Stepankova H.; Novak P.; Biało I.; Honig J. M. Effect of low doping on the Verwey transition in magnetite single crystals: Mössbauer spectroscopy and x-ray diffraction. Phys. Rev. B 2018, 98, 125138 10.1103/PhysRevB.98.125138.

[ref52] Kim T.; Sim S.; Lim S.; Patino M. A.; Hong J.; Lee J.; Hyeon T.; Shimakawa Y.; Lee S.; Attfield J. P.; Park J. G. Slow oxidation of magnetite nanoparticles elucidates the limits of the Verwey transition. Nat. Commun. 2021, 12, 6356 10.1038/s41467-021-26566-4. 34737260 PMC8568917

[ref53] Pachoud E.; Cumby J.; Perversi G.; Wright J. P.; Attfield J. P. Site-selective doping of ordered charge states in magnetite. Nat. Commun. 2020, 11, 1671 10.1038/s41467-020-15504-5. 32245968 PMC7125154

[ref54] Perversi G.; Cumby J.; Pachoud E.; Wright J. P.; Attfield J. P. The Verwey structure of a natural magnetite. Chem. Commun. 2016, 52, 4864–4867. 10.1039/C5CC10495E. 26908195

[ref55] Attfield J. P. Orbital molecules in electronic materials. APL Mater. 2015, 3, 041510 10.1063/1.4913736.

[ref56] Lavina B.; Dera P.; Kim E.; Meng Y.; Downs R. T.; Weck P. F.; Sutton S. R.; Zhao Y. S. Discovery of the recoverable high-pressure iron oxide Fe4O5. Proc. Natl. Acad. Sci. U.S.A. 2011, 108, 17281–17285. 10.1073/pnas.1107573108. 21969537 PMC3198347

[ref57] Bykova E.; Dubrovinsky L.; Dubrovinskaia N.; Bykov M.; McCammon C.; Ovsyannikov S. V.; Liermann H. P.; Kupenko I.; Chumakov A. I.; Ruffer R.; Hanfland M.; Prakapenka V. Structural complexity of simple Fe2O3 at high pressures and temperatures. Nat. Commun. 2016, 7, 10661 10.1038/ncomms10661. 26864300 PMC4753252

[ref58] Ovsyannikov S. V.; Bykov M.; Bykova E.; Kozlenko D. P.; Tsirlin A. A.; Karkin A. E.; Shchennikov V. V.; Kichanov S. E.; Gou H.; Abakumov A. M.; Egoavil R.; Verbeeck J.; McCammon C.; Dyadkin V.; Chernyshov D.; van Smaalen S.; Dubrovinsky L. S. Charge-ordering transition in iron oxide Fe4O5 involving competing dimer and trimer formation. Nat. Chem. 2016, 8, 501–508. 10.1038/nchem.2478. 27102685

[ref59] Ovsyannikov S. V.; Bykov M.; Bykova E.; Glazyrin K.; Manna R. S.; Tsirlin A. A.; Cerantola V.; Kupenko I.; Kurnosov A. V.; Kantor I.; Pakhomova A. S.; Chuvashova I.; Chumakov A. I.; Ruffer R.; McCammon C.; Dubrovinsky L. S. Pressure tuning of charge ordering in iron oxide. Nat. Commun. 2018, 9, 4142 10.1038/s41467-018-06457-x. 30297769 PMC6175922

[ref60] Ovsyannikov S. V.; Bykov M.; Medvedev S. A.; Naumov P. G.; Jesche A.; Tsirlin A. A.; Bykova E.; Chuvashova I.; Karkin A. E.; Dyadkin V.; Chernyshov D.; Dubrovinsky L. S. A Room-Temperature Verwey-type Transition in Iron Oxide, Fe5O6. Angew. Chem., Int. Ed. 2020, 59, 5632–5636. 10.1002/anie.201914988. PMC7154779 31899577

[ref61] Angst M.; Hermann R. P.; Schweika W.; Kim J.-W.; Khalifah P.; Xiang H. J.; Whangbo M.-H.; Kim D.-H.; Sales B. C.; Mandrus D. Incommensurate charge order phase in Fe2OBO3 due to geometrical frustration. Phys. Rev. Lett. 2007, 99, 256402 10.1103/PhysRevLett.99.256402. 18233535

[ref62] Hearne G. R.; Sibanda W. N.; Carleschi E.; Pischedda V.; Attfield J. P. Pressure-induced suppression of charge order and nanosecond valence dynamics in Fe2OBO3. Phys. Rev. B 2012, 86, 195134 10.1103/PhysRevB.86.195134.

[ref63] Diguet G.; Hearne G. R.; Sibanda W. N.; Carleschi E.; Musyimi P.; Pischedda V.; Attfield J. P. Wigner-Mott insulator-to-insulator transition at pressure in charge-ordered Fe2OBO3. Phys. Rev. B 2014, 89, 035132 10.1103/PhysRevB.89.035132.

[ref64] Delacotte C.; Hüe F.; Breard Y.; Hebert S.; Perez O.; Caignaert V.; Greneche J. M.; Pelloquin D. Structural Transition at 360 K in the CaFe5O7 Ferrite: Toward a New Charge Ordering Distribution. Inorg. Chem. 2014, 53, 10171–10177. 10.1021/ic5011456. 25203604

[ref65] Hong K. H.; McNally G. M.; Coduri M.; Attfield J. P. Synthesis, crystal structure, and magnetic properties of MnFe3O5. Z. Anorg. Allg. Chem. 2016, 642, 1355–1358. 10.1002/zaac.201600365.

[ref66] Hong K. H.; Arevalo-Lopez A. M.; Coduri M.; McNally G. M.; Attfield J. P. Cation, magnetic, and charge ordering in MnFe3O5. J. Mater. Chem. C 2018, 6, 3271–3275. 10.1039/C8TC00053K. PMC6003543 30009028

[ref67] Cassidy S. J.; Orlandi F.; Manuel P.; Clarke S. J. Single phase charge ordered stoichiometric CaFe3O5 with commensurate and incommensurate trimeron ordering. Nat. Commun. 2019, 10, 5475 10.1038/s41467-019-13450-5. 31792221 PMC6889228

[ref68] Hong K. H.; Arevalo-Lopez A. M.; Cumby J.; Ritter C.; Attfield J. P. Long range electronic phase separation in CaFe_3_O_5_. Nat. Commun. 2018, 9, 2975 10.1038/s41467-018-05363-6. 30061576 PMC6065443

[ref69] Hong K. H.; Solana-Madruga E.; Hakala B. V.; Patino M. A.; Manuel P.; Shimakawa Y.; Attfield J. P. Substitutional tuning of electronic phase separation in CaFe3O5. Phys. Rev. Mater. 2021, 5, 024406 10.1103/PhysRevMaterials.5.024406.

